# *Vaccinium ashei* leaves extract alleviates insulin resistance via AMPK independent pathway in C2C12 myotube model

**DOI:** 10.1016/j.bbrep.2018.05.003

**Published:** 2018-05-22

**Authors:** Masao Yamasaki, Kensaku Hamada, Kento Fujii, Kazuo Nishiyama, Yumi Yamasaki, Hiroyuki Tari, Kaori Araki, Teruaki Arakawa

**Affiliations:** a Faculty of Agriculture, University of Miyazaki, 1-1 Gakuenkibanadai-nishi, Miyazaki 889-2192, Japan; b Faculty of Regional Innovation, University of Miyazaki, 1-1 Gakuenkibanadai-nishi, Miyazaki 889-2192, Japan; c Bizen Chemical Company Limited, 363 Tokudomi, Akaiwa 709-0716, Japan

**Keywords:** Adipocyte, Inflammation, Nitric oxide, *Vaccinium ashei*

## Abstract

Blueberry (*Vaccinium ashei*) leaf has recently attracted attention as an anti-obesity food component. In this study, we examined the effects of blueberry leaf extract (BLEx) on insulin signaling in C2C12 differentiated myoblasts. The results showed that BLEx promotes the intracellular uptake of 2-[*N*-(7-Nitrobenz-2-oxa-1,3-diazol-4-yl)amino]−2-deoxy-D-glucose (2-*N*BDG) and phosphorylation of Akt under insulin stimulation. In addition, pretreatment with BLEx ameliorated TNF-α-nduced insulin resistance with regard to 2-NBDG uptake and Akt phosphorylation. Moreover, BLEx prevented the TNF-α−induced activation of JNK and NF-kB pathways and phosphorylation of IRS-1 at serine residue. BLEx failed to induce phosphorylation of AMPK as well as did not prevent the restoration of 2-NBDG uptake under TNF-α–induced insulin resistance. Overall, skeletal muscle is a putative target for the anti-diabetic effect of BLEx by amelioration of insulin resistance.

## Highlights

• Blueberry leaf extract (BLEx) promotes the intracellular uptake of 2-NBDG.
• BLEx prevented the TNF-α−induced insulin resistance.
• BLEx failed to activate AMPK pathway.
• Skeletal muscle is putative target of the BLEx for its anti-diabetic effect.

## 1 Introduction

Prevention and alleviation of obesity related life-style diseases are emerging pertinent issues all over the world. Vigorous functional food studies have been performed and various promising materials and food components for anti-obesity related diseases have been proposed. Some of them include very popular ingredients such as green tea in Japan, whereas efficacies of roots, flowers, and leaves in rare plants, which are often passed down from the older generation as folk medicine, are attracting increasing attention.

Blueberry (*Vaccinium ashei*) is native to southeastern America and belongs to the *Ericaceae* plant family. Recent studies have revealed that not only the fruits but also blueberry leaves have various beneficial effects on human health. Especially, anti-diabetic effects of blueberry fruit or its juice have been reported [1]. Meanwhile, blueberry leaf infusion has been used as a folk medicine for lifestyle-related diseases in Europe, although scientific evidence proving its benefits has not been fully established. Sufficient evidence for the safety of blueberry leaf infusion has been established in humans and blueberry leaf is now processed into a beverage and is available commercially in Japan. According to these cultural and commercial backgrounds, blueberry leaf infusion could be developed as a functional food. Blueberry infusion has slight sour and astringent taste because of the presence of organic acid such as quinic acid and is rich in various polyphenols, especially rutin, chlorogenic acid, and proanthocyanidins [2], [3]. Although these are not necessarily characteristic components in leaves, leaf tissues have higher oxygen radical absorbance capacity with higher total phenolic content than in fruit tissues [4]. It has recently been reported that blueberry leaf extract has an inhibitory effect on angiotensin-converting enzyme activity and reduces plasma glucose and triglyceride in streptozotocin-diabetic rats [5], [6]. Our study showed that dietary blueberry leaf hot water extract (BLEx) ameliorated abnormal blood glucose level in high-fat, high-sucrose induced obese mice, indicating its preventive effect on type II diabetes [7]. However, the molecular mechanism of the anti-diabetic effect of BLEx is unclear.

It is of importance that accumulation of scientific evidences with detail molecular mechanism(s) for the safe and effective application of BLEx as functional materials. Here, we focused on the effect of BLEx on the glucose metabolism in skeletal muscle that is the highest glucose consuming organ, because, the effect of BLEx on the glucose metabolism in skeletal muscle is totally unknown. To study the direct effect on the skeletal muscle glucose metabolism, C2C12 cells have been utilized as a model of myotube in vitro because the property of glucose metabolism has been well-characterized and standard protocol for the myoblast to myotube has been well-established. Here, we preliminary confirmed the glucose uptake activity of this cells after the differentiation to myotube. Additionally, it is well-known that sensitivity of skeletal muscle cells to insulin is deteriorated (insulin resistance) in the patients with type II diabetes resulting in downregulation of intracellular glucose uptake and postprandial increase of blood glucose. Elucidation of molecular target of BLEx on the skeletal muscle may give information to reveal the active component of BLEx in further study.

## 2 Materials and methods

### 2.1 Materials

Hot water BLEx extract was procured from Bizen Chemical Co. Ltd (Okayama, Japan). Briefly, blueberry powder was extracted in 16 parts of hot water (95–100 °C) for 30 min twice. Next, the extract was filtered, sterilized by heat, dried with spray dryer, and converted into powder form. The composition of BLEx was analyzed by Japan Food Research Laboratories (Tokyo, Japan) and shown in Table 1. In addition, BLEx contained 368 mg eq. procyanidin B1/g proanthocyanidin (MASIS Inc, Food & Drug Nano Analysis, Aomori, Japan), 73.0 mg/g chlorogenic acid, and 187 mg/g quinic acid (Japan Food Research Laboratories). Total polyphenol content was 403 mg eq. tannic acid/g. Antibodies for AMPKα, phospho-AMPKα (Thr172), IkB-α, phospho IkB-α (Ser32/36), NF-κB p65, phospho-NF-κB p65 (Ser536), SAPK/JNK, phospho-SAPK/JNK, Akt (pan), phospho-Akt (ser473), IRS-1, HRP conjugated anti-mouse IgG, and HRP conjugated anti- rabbit IgG were purchased from Cell Signaling Technology (Danvers, MA). Antibodies for β-actin (clone AC-15) and phospho-IRS-1 (ser307) were purchased from Sigma (St. Louis, MO) and Cusabio Biotech (College Park, MD), respectively. 5-Aminoimidazole-4-carboxamide-1-β-D-ribofuranoside (AICAR) and compound C were purchased from Sigma and Wako (Osaka, Japan), respectively.

**Table 1:** Composition of BLEx.

Component (g / 100 g)	
Moisture	5.1
Protein	1.1
Fat	0.4
Ash	5.6
Others	87.8
Polyphenol: 403 mg eq. tannic acid/g	
Chlorogenic acid 73.0 mg/g	
Quinic acid 187 mg/g	
Proanthocyanidin 368.8 mg eq. procyanidinB1/g	

### 2.2 Cell culture

**Fig. 1:**
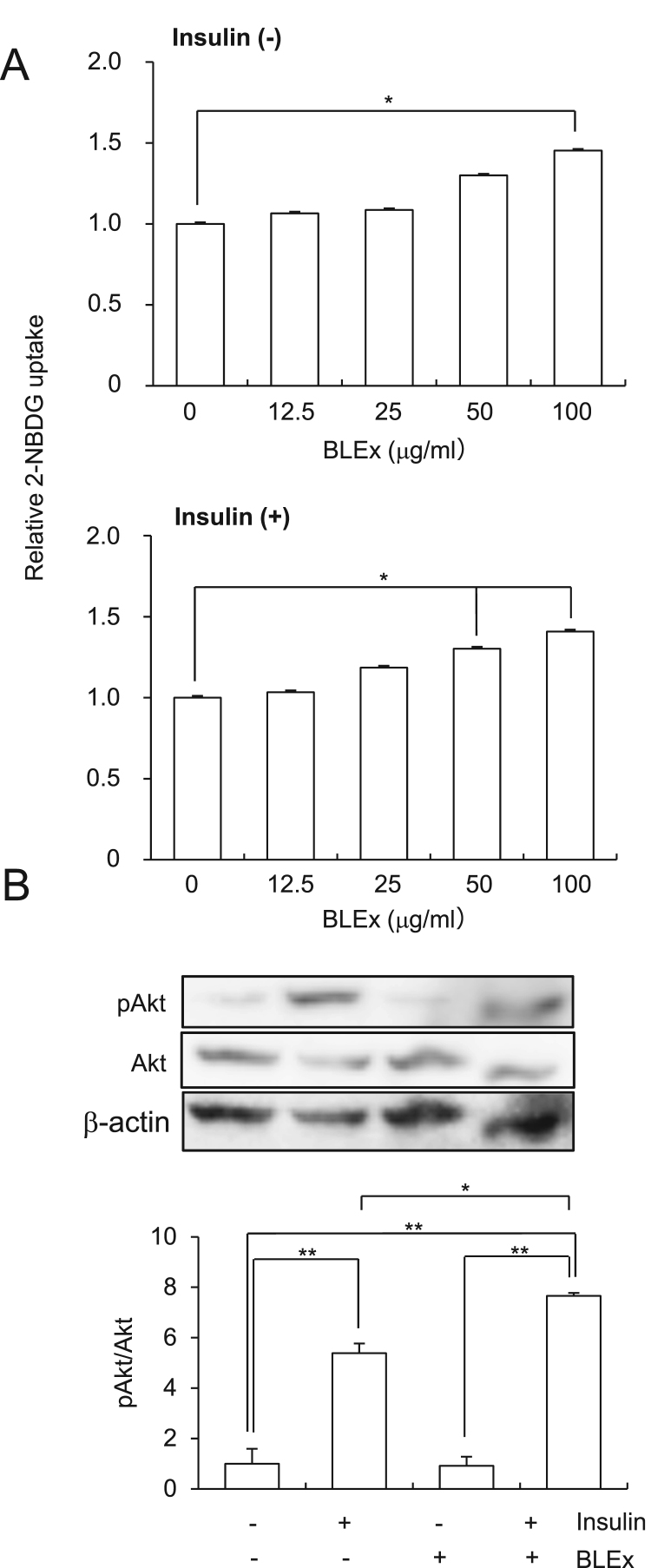
Effect of BLEx on the insulin response in C2C12 myotubes. Data are represented as mean ± SE for three independent experiments and asterisk mark(s) shows the statistically significant difference at *, **p < 0.05 and 0.01, respectively. C2C12 cells were treated with indicated concentrations of BLEx for 24 h and subsequently treated with or without insulin (100 nM) for 30 min. BLEx used in B was 100 μg/mL. A; 2-NBDG incorporation, B; Akt phosphorylation.

C2C12 cells were purchased from Riken BioResource Center (Tsukuba, Japan). These cells were maintained in DMEM supplemented with 10% fetal bovine serum (FBS) containing penicillin-streptomycin-amphotericin. Cells were subcultured twice a week. For the differentiation to myotube, cells were seeded at 5.0 × 10^3^ cells/cm^2^ and cultured for 48 h to confluency. Subsequently, cells were cultured with 2% horse serum for four days to terminate the differentiation. Further, cells were pretreated with 0–100 μg/mL BLEx (especially 100 μg/mL in Figs. 1B and 5C) with or without tumor necrosis factor (TNF)-α (10 ng/mL) for 24 h before 2-NBDG treatment for the analysis of glucose uptake and insulin signaling related proteins by western blot.

### 2.3 Glucose uptake

For the analysis of 2-[*N*-(7-Nitrobenz-2-oxa-1,3-diazol-4-yl)amino]−2-deoxy-D-glucose (2-NBDG, Cayman Chemical, MI) uptake, cells were seeded into 96-well black plates (Thermo Fisher Scientific Inc, MA) to undergo differentiation according to the above described procedure. Next, the supernatants were removed and treated with 100 nM insulin in Hank's buffered saline for 30 min followed by treatment with 50 μM 2-NBDG for 30 min. Compound C, an inhibitor for AMPK pathway, was added 30 min before BLEx treatment. At the end of the culture period, cells were washed with PBS and 2-NBDG uptake was evaluated by measuring fluorescence intensity (Ex/Em = 485/535 nm).

### 2.4 Western blotting

To analyze cellular proteins, cells were cultured in 6-well plates and cellular proteins were detected by western blot analysis. At the end of the culture period, cells were lysed in 50 mM Tris-HCl (pH 7.5) containing 150 mM NaCl, 2% Triton X-100, 2 mM EDTA, 50 mM NaF, 30 mM Na_4_P_2_O_7_ with protease inhibitor cocktail (Nacalai Tesque, Kyoto, Japan). Protein concentrations were measured using the BCA protein assay reagent (Thermo Fisher Scientific) and 10 μg protein was subjected to the electrophoresis to adjust the loading amount of the protein. For further verification of loading control, both non-phosphorylated and phosphorylated proteins were detected. Lysates were separated by electrophoresis on a 10% SDS-polyacrylamide gel and susbequently transferred to PVDF Hybond-P membrane (Thermo Fisher Scientific). Blocking was performed using 5% defatted milk or bovine serum albumin (for the detection of phosphorylated proteins) in Tris-buffered saline with 0.1% Tween-20 (TTBS), and antibodies were diluted in Can Get Signal solutions 1 and 2 (Toyobo, Tokyo, Japan). The membrane was washed with TTBS after each antibody binding reaction. Detection of each protein was performed using the Clarity western ECL substrate (Bio-Rad, Hercules, CA) and the band intensity was quantified using ImageJ software. The data shows representative blot patterns and mean values were determined for at least three independent experiments.

### 2.5 Statistical analysis

Statistical analysis was done with 4 Steps Statcel2 software (OMS publishing, Saitama, Japan). The data were analyzed by one-way analysis of variance and post-hoc test (Turkey Kramer multiple comparisons). Differences at *P* < 0.05 were considered significant.

## 3 Results

Firstly, the response to insulin stimulation of differentiated C2C12 cells with respect to 2-NBDG uptake were evaluated (Fig. 1A) and data are shown as relative values to the control with (lower panel) or without (upper panel) insulin, respectively. There is no significant difference in the base line of 2-NBDG uptake between insulin (-) and insulin (+). The cells were pretreated with BLEx for 24 h followed by insulin stimulation. The results showed that BLEx promoted 2-NBDG uptake irrespective of insulin stimulation and this effect was observed at lower doses when cells were treated with insulin. As Fig. 1A implied a promotive effect of BLEx on insulin response, Akt phosphorylation, one of the major events in insulin signaling, was evaluated. Fig. 1B showed that insulin, but not BLEx, apparently promoted Akt phosphorylation and BLEx synergistically promoted insulin induced Akt phosphorylation.

As BLEx tended to promote insulin response, we attempted to understand whether BLEx could ameliorate the impaired insulin signaling induced by TNF-α which is a representative adipokine that induces insulin resistance in muscle cells. The results revealed that TNF-α significantly prevented insulin induced 2-NBDG uptake and Akt phosphorylation, thereby verifying the insulin resistance. Interestingly, BLEx at 100 μg/mL completely restored the 2-NBDG uptake and Akt phosphorylation under TNF-α treatment. It has been reported that TNF-α induced insulin resistance in skeletal muscles is attributed to the induction of IRS-1 phosphorylation on serine residue followed by the deterioration of insulin signaling. As shown in Fig. 3, TNF-α strikingly promoted IRS-1 serine phosphorylation whereas BLEx clearly suppressed the action of TNF-α.

**Fig. 2:**
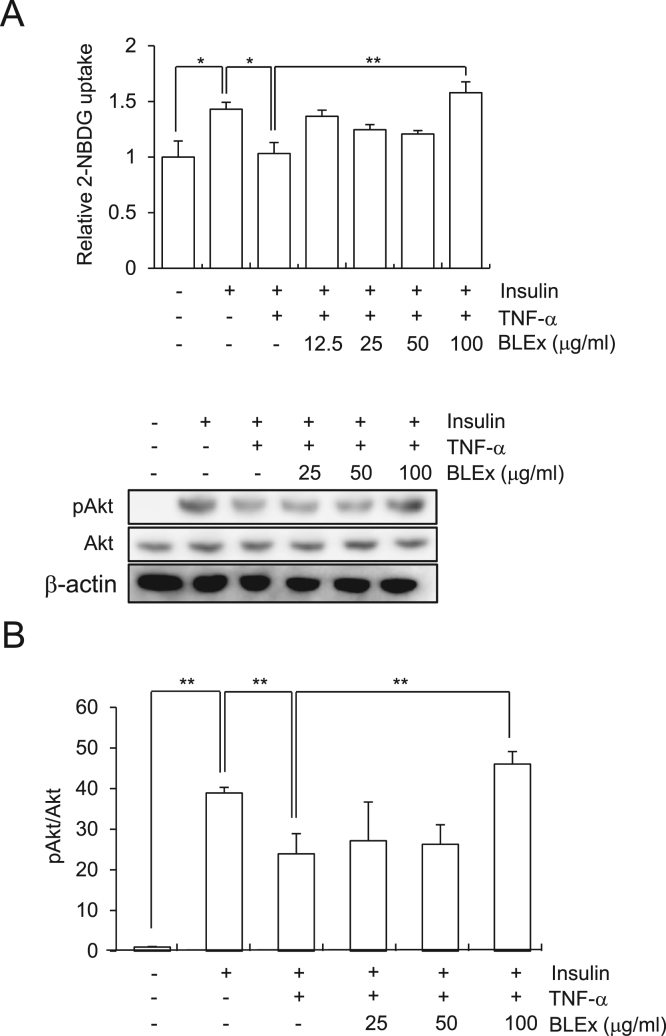
Effect of BLEx on the TNF-α induced insulin resistance in C2C12 myotubes. Data are represented as mean ± SE for three independent experiments and asterisk mark(s) shows the statistically significant difference at *, **p < 0.05 and 0.01, respectively. C2C12 cells were treated with indicated concentrations of BLEx with 10 ng/mL TNF-α for 24 h and further treated with or without insulin (100 nM) for 30 min. A; 2-NBDG incorporation, B; Akt phosphorylation.

**Fig. 3:**
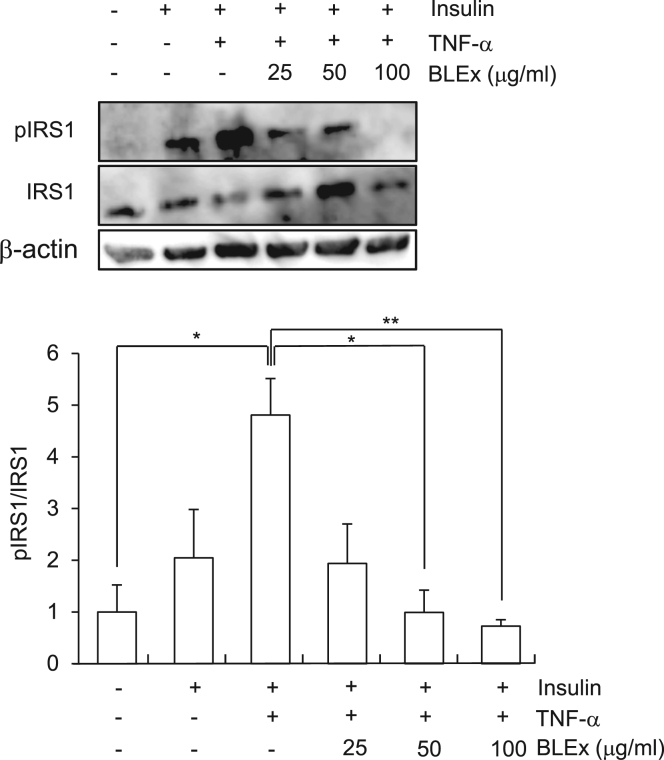
Effect of BLEx on the TNF-α induced IRS-1 phosphorylation at serine 307 in C2C12 myotubes. Data are represented as mean ± SE for three independent experiments and asterisk mark(s) shows the statistically significant difference at *, **p < 0.05 and 0.01, respectively. C2C12 cells were treated with indicated concentrations of BLEx with 10 ng/mL TNF-α for 24 h and further treated with or without insulin (100 nM) for 30 min.

**Fig. 4:**
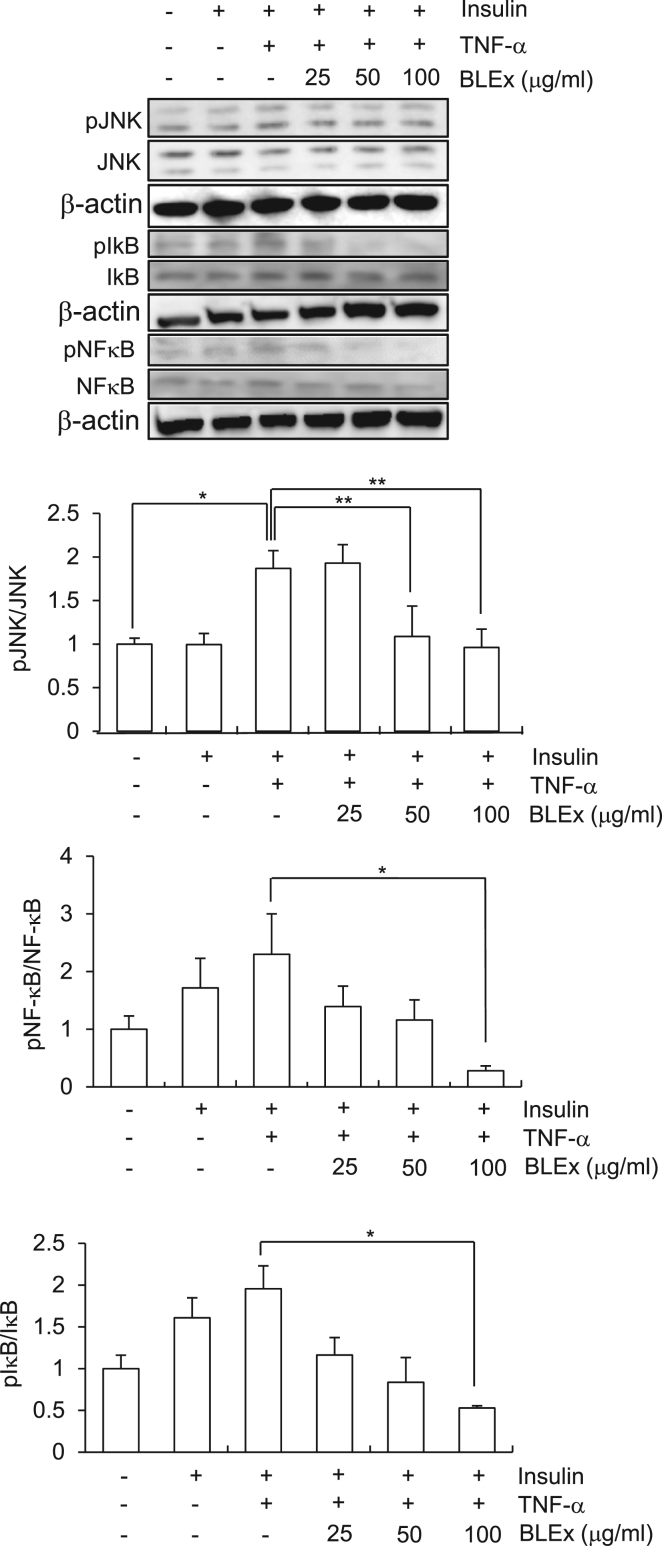
Effect of BLEx on the TNF-α induced activation of JNK and NF-κB pathway in C2C12 myotubes. Data are represented as mean ± SE for three independent experiments and asterisk mark(s) shows the statistically significant difference at *, **p < 0.05 and 0.01, respectively. C2C12 cells were treated with indicated concentrations of BLEx with 10 ng/mL TNF-α for 24 h and further treated with or without insulin (100 nM) for 30 min.

The engagement of TNF-α with its receptor initiates a variety of intracellular signaling events and activation of JNK and NF-κB pathways are representative events of this phenomenon. We evaluated the activation of these signaling pathways by assaying the phosphorylation of JNK, IkB, and NF-kB p65. TNF-α increased the phosphorylation of these proteins and 100 μg/mL BLEx significantly suppressed the phosphorylation of all these proteins (Fig. 4A, B, C).

**Fig. 5:**
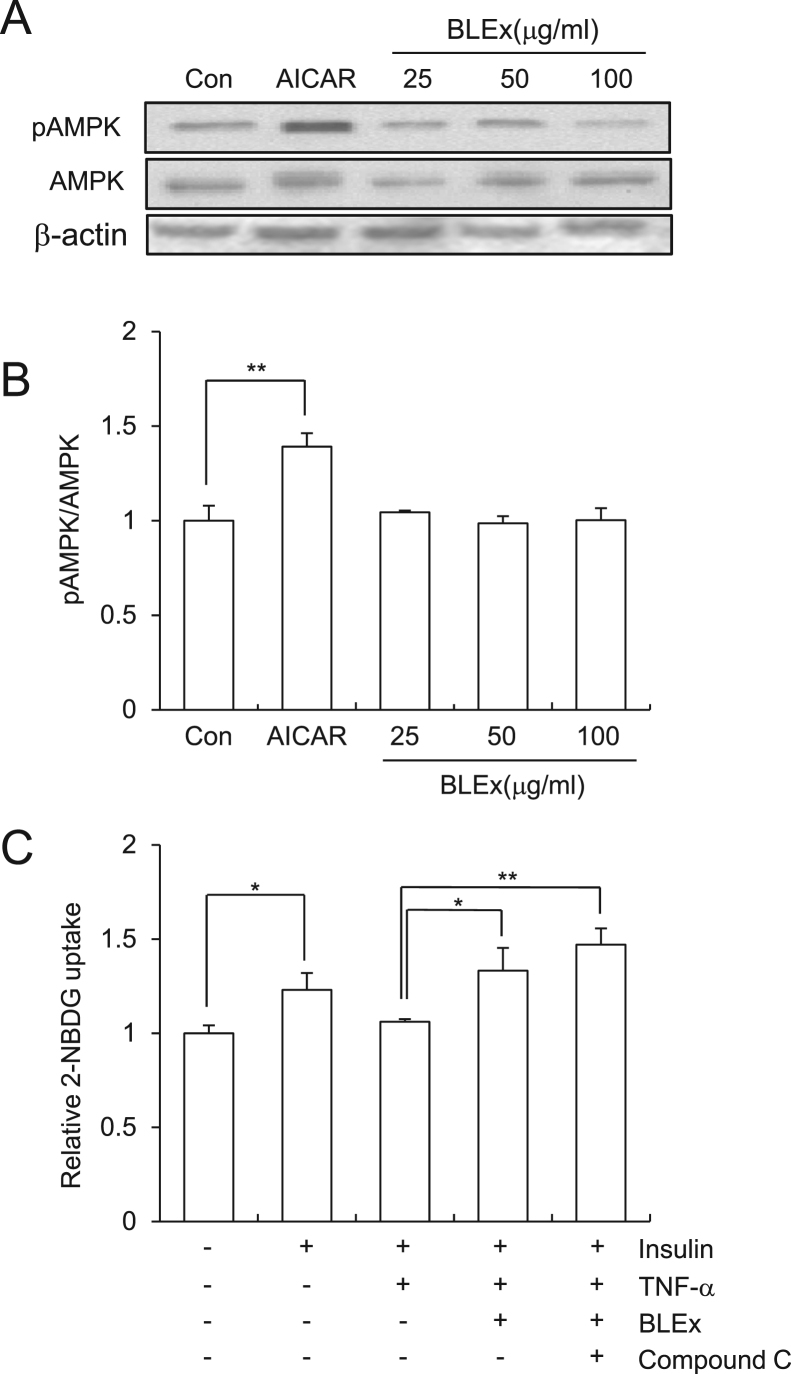
Evaluation of the involvement of AMPK pathway in the action of BLEx in C2C12 myotubes. Data are represented as mean ± SE for three independent experiments and asterisk mark(s) shows the statistically significant difference at *, **p < 0.05 and 0.01, respectively. C2C12 cells were treated with indicated concentrations of BLEx with 10 ng/mL TNF-α for 24 h and further treated with or without insulin (100 nM) for 30 min. BLEx used in C was 100 μg/mL.

Finally, we evaluated the effect of BLEx on the AMPK pathway because activation of AMPK is known to elicit insulin independent glucose uptake and to ameliorate insulin resistance in skeletal muscles. In our study, AICAR, an AMPK activator, but not BLEx, robustly induced phosphorylation of AMPK. Moreover, compound C, an inhibitor of AMPK, failed to prevent the promoting effect of BLEx on 2-NBDG uptake under TNF-α treatment (Fig. 5).

## 4 Discussion

Insulin binding to its cognate receptor initiates signaling resulting in the membrane translocation of Glut-4, followed by increase in glucose incorporation. Further, insulin promoted 2-NBDG incorporation (Fig. 2). Treatment with BLEx promoted 2-NBDG incorporation at 100 μg/mL and co-treatment of BLEx at 50 μg/mL and insulin synergistically promoted glucose incorporation. Activation of insulin receptor evokes phosphorylation of IRS-1 on tyrosine residue followed by the activation of PI3K/Akt pathway [8], and our data showed apparent activation of Akt phosphorylation by insulin. Intriguingly, BLEx promoted phosphorylation of Akt when cells were co-stimulated with insulin, however no effect was observed in the absence of insulin stimulation. These data indicate that BLEx promote insulin signaling without insulin-mimetic activity. Proanthocyanidins (PAC) are representative polyphenol compounds in BLEx and are considered to play a pivotal role in the preventive effect on lipid accumulation in the liver [9], [10]. PACs abundantly occur in various edible plants and some reports showed their insulin-mimetic activity whereas BLEx did not demonstrate this property in this study [11], [12]. Adipocytes have been often used as the target of insulin in previous reports, however, Pinent showed insulin-mimetic effect of grape seed-derived PACs on the differentiated myotube [13]. Therefore, the mode of the action of BLEx might be possibly different from other PAC-rich materials.

Deterioration in insulin signaling or insulin resistance in skeletal muscle cells is one of hallmark features of type II diabetes. Inflammatory adipokine TNF-α is one of the factors responsible for this insulin resistance through activation of TNF receptor-JNK signaling pathway, resulting in the phosphorylation of serine residue of IRS-1 [14], [15]. Serine phosphorylation of IRS-1 results in substantial impairment of downstream insulin signaling such as activation of PI3K/Akt pathway and Glut-4 translocation [8], [16]. The present data in C2C12 cells also recapitulated the impairment of insulin signaling by TNF-α treatment in terms of suppression of 2-NBDG uptake. Therefore, alleviation of impaired insulin signaling induced by TNF-α treatment can be a useful target for the screening of anti-diabetic materials [17]. As BLEx treatment restored the 2-NBDG uptake under TNF-α treatment, BLEx is a promising material that is capable of promoting insulin signaling and alleviating insulin resistance.

**Fig. 6:**
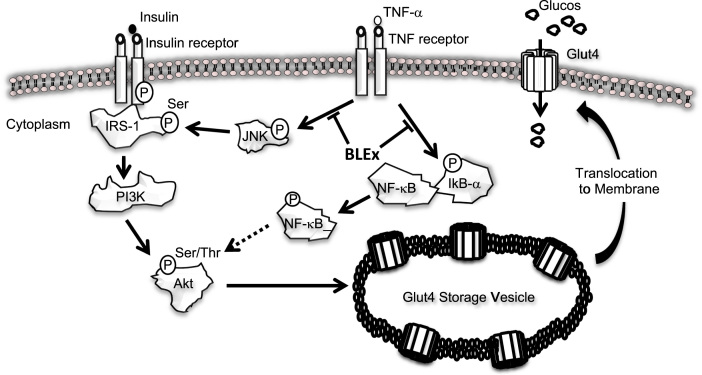
Speculated mechanism in BLEx involving alleviating effect of insulin resistance.

Although TNF-α elicits insulin resistance in the hepatocytes, adipocytes, and activates skeletal muscle cells and phosphorylation of IRS-1 on serine residue is a common event, detailed signaling pathway is different in these cells. Activation of MAPK pathways such as JNK, ERK, and p38 accompanied with IKK activation are the hallmark events after TNF-α treatment [18] and our results are consistent with the activation of JNK and IKK detected indirectly by phosphorylation of IkB-α. Our results clearly showed that BLEx suppressed all these activation trends induced by TNF-α. On the other hand, it has been reported that IKK is responsible for the phosphorylation of IRS-1, however, inhibition of JNK pathway failed to normalize the IKK activity [14]. These data suggest BLEx targets the molecule(s) that is part of upstream signaling and orchestrates both IKK and JNK pathways (Fig. 6).

Independent of insulin action, activation of AMPK pathway promotes Glut-4 translocation and the following glucose uptake in skeletal muscle cells [19], [20], [21], [22]. AICAR is a well-known activator of AMPK pathway and is capable of enhancing insulin response in skeletal muscle cells [23] and the present data shown in Fig. 5 is consistent with the previous report of AMPK activation. Therefore, AMPK pathway is attracting attention in the context of enhancement of insulin sensitivity and alleviation of insulin resistance and it is reasonable to evaluate the effect of BLEx on the AMPK pathway. Alleviation of insulin resistance by phenolic compounds has been attracting attention and AMPK is a promising candidate as a molecular target of food components [24]. Among them, tea catechin, chlorogenic acid, and grape proanthocyanidin activate AMPK signaling to suppress insulin resistance [25], [26]. As BLEx contained chlorogenic acid and proanthocyanidin as major polyphenolic compounds, we assumed that AMPK was a plausible candidate as the molecular target of its anti-diabetic effect. However, results clearly showed that BLEx failed to activate AMPK phosphorylation and inhibition of AMPK did not abrogate the action of BLEx, indicating an AMPK independent mechanism. As shown in Fig. 1, BLEx promoted 2-NBDG uptake during insulin stimulation without TNF-α treatment, indicating existence of another additional molecular mechanism independent of TNF-α related signaling. For instance, cocoa polyphenol inhibits insulin receptor kinase activity and the subsequent insulin signaling [27].

Our previous study revealed that BLEx is a promising material for the prevention of type II diabetes and this study implies the involvement of amelioration of insulin resistance in skeletal muscles as an anti-diabetic effect of BLEx.

## Author disclosure statement

No competing financial interest exists.
